# The Development of an Experimental Multiple Serogroups Vaccine for *Neisseria meningitidis*

**DOI:** 10.1371/journal.pone.0079304

**Published:** 2013-11-14

**Authors:** Valerian B. Pinto, Robert Burden, Allyn Wagner, Elizabeth E. Moran, Che-Hung Lee

**Affiliations:** 1 Division of Bacterial and Rickettsial Diseases, Walter Reed Army Institute of Research (WRAIR), Silver Springs, Maryland, United States of America; 2 Center for Biologics Evaluation and Research, Food and Drug Administration (FDA), Bethesda, Maryland, United States of America; Instituto Butantan, Brazil

## Abstract

A native outer membrane vesicles (NOMV) vaccine was developed from three antigenically diverse strains of *Neisseria meningitidis* that express the L1,8, L2, and L3,7 lipooligosaccharide (LOS) immunotypes, and whose *synX*, and *lpxL1* genes were deleted.. Immunogenicity studies in mice showed that the vaccine induced bactericidal antibody against serogroups B, C, W, Y and X *N. meningitidis* strains. However, this experimental NOMV vaccine was not effective against serogroup A *N. meningitidis* strains. *N. meningitidis* capsular polysaccharide (PS) from serogroups A, C, W and Y were effective at inducing bactericidal antibody when conjugated to either tetanus toxoid or the fHbp1-fHbp2 fusion protein fHbp(1+2). The combination of the NOMV vaccine and the *N. meningitidis* serogroup A capsular polysaccharide (MAPS) protein conjugate was capable of inducing bactericidal antibodies against a limited number of *N. meningitidis* strains from serogroups A, B, C, W, Y and X tested in this study.

## Introduction

Among thirteen isolated meningococcal serogroups, strains belonging to serogroups A, B, C, W and Y account for the majority of meningococcal disease in humans. Capsular polysaccharides have been used successfully as vaccines to prevent *N. meningitidis* serogroups A, C, Y and W infections [1]–[4], but are not capable of serogroup cross protection. Recent cases of meningococcal disease caused by serogroups X [5]–[8] and B, for which there are no FDA-approved vaccines, and the ability of *N. meningitidis* to switch capsules [9]–[10], demonstrate the limited utility of vaccines based solely on capsular antigens. Moreover, the serogroup B capsular polysaccharide (PS) is a polysialic acid with an α2→8 glycosidic linkage, which is similar to the PS structure expressed in certain human tissues [11], thus making it a poor immunogen. Therefore, efforts to develop a group B meningococcal vaccine have largely focused on the subcapsular antigens.

Since subcapsular antigens are not necessarily serogroup specific, it is quite likely that the vaccines currently being developed to combat serogroup B *N. meningitidis* infections may also be effective against other serogroups. Currently at least five subcapsular vaccines are being developed for broad protection against serogroup B *N. meningitidis*: 1) the PorA based vaccines [12]–[13]; 2) an LOS based vesicle vaccine [14]; 3) the 4CMenB vaccine containing Neisserial adhesin A (NadA), Factor H-binding protein variant 1 (fHbp-1), Neisserial heparin-binding antigen (NHBA), GNA 2091, GNA 1030 and OMV from the New Zealand epidemic strain [15]; 4) The bi-valent fHbp subfamily A and subfamily B vaccine [16], and 5) The recently described native outer membrane vesicle (NOMV) vaccines [17]. All these serogroup B *N. meningitidis* vaccines, which are based on the subcapsular antigens, have the added benefit of generating antibodies to antigens that are also present in other serogroups. The NOMV vaccine and the vaccines containing fHbp have been shown to be protective against strains of *N. meningitidis* from other serogroups [18]–[19]. Thus a combination of subcapsular antigens and capsular polysaccharide conjugates may result in the generation of a multi-serogroup *N. meningitidis* vaccine.

In this communication we report the development of a multi-serogroup *N. meningitidis* vaccine which is a combination of a vesicle vaccine (NOMV) and a capsular conjugate. The strains used to make the NOMV vaccine were based on the assumption that vaccines containing L2 and L3,7 LOS would cover 80% of the pathogenic serogroup B N. meningitidis strains [20], and that approximately 60% of the disease-causing serogroup B N. meningitidis strains in the MLST database belong to either ST 41/44, ST-11 or ST32 MLST complex [21]. The NOMV along with serogroup A capsule conjugated to either tetanus toxoid or fHbp1 and fHbp2 fusion protein fHbp(1+2) induced a protective response against the limited number of *N. meningitidis* strains from serogroups A, B, C, W, Y and X tested in this study.

## Materials and Methods

### Ethics Statement

This research was conducted in compliance with the Animal Welfare Act and other federal statutes and regulations relating to animals and experiments involving animals and adheres to principles stated in the *Guide for the Care and Use of Laboratory Animals*, NRC Publication, 1996 edition. The animal care and use protocol number 1999-28 was approved by the IACUC/ethics committee of CBER/FDA.

### Bacterial Strains used to make the Vaccine, and used as Targets in the Bactericidal Assay

The bactericidal target strains used for serogroups B, C, Y and W were mostly isolated from U.S. Army personnel prior to routine vaccination with the tetravalent polysaccharide vaccine (about 1982). The four serogroup A target strains were obtained from other investigators and originated in Egypt, Africa, Finland, and Germany. The serogroup X strains were obtained from the Naval Medical Unit-3 (NAMRU-3) in 2009, and were isolates from a meningococcal meningitis outbreak in western Kenya [8]. Only meningococcal strains for which human complement was available at the WRAIR were used in the bactericidal assay.

### Genetic Modifications of the Bacterial Strains used to make the Vaccine

The only two modifications made to all three vaccine strains were deletion of the capsule by disrupting the *synX* gene with the Kan^r^ resistance marker and changing acylation of the LOS from hexa-acylation to a penta-acylation by disrupting the *lpxL1* gene with the Tet^r^ marker. The two plasmids used for the modification, pMn5 (Tet^r^) and pZero-synX-Kan (Kan^r^) were kindly provided by Dr. Wendell D. Zollinger. Transformation was performed by adding 10 µl of plasmid DNA to 5–10 colonies of overnight cultures plated on 1 cm^2^ area of a GC agar plate and incubated for 4–6 hours at 37^o^C in 5% CO_2_. The cells were suspended in 1.0 ml of Mueller-Hinton broth and plated on GC agar plates supplemented with the appropriate antibiotic. Transformation was done sequentially: first the bacteria were transformed with the plasmid pMn5 (Tet^r^), and selected on GC-tetracycline plates (tetracycline concentration of 5 µg/ml), followed by transformation by pZero-synX-Kan (Kan^r^), and selected on GC-Kanamycin plates (Kanamycin concentration of 5 µg/ml). The *lpxL1* knockout was confirmed by PCR using primers htrb(f) 5′-GACCGTCTGAAACGGATGAAATTTATATTTTTTGTAC-3′ and htrb(r) 5′-TCAGTAAAAATCGGGGCTGCCTTCCGG-3′. PCR was carried out in 50 µl reaction volumes (45 µl Platinum Blue PCR Supermix (Invitrogen), 2 µl genomic DNA, 1.5 µl each primer. The PCR conditions were as follows: an initial step of 94°C for 5 min, followed by 30 cycles of 94°C for 1 min, 60°C for 1.5 min, 72°C for 4 min, and a final step of 72°C for 10 min. The *synX* knockout was confirmed by PCR using primers SynX5 (f) 5′-CCGGTCGACGACCGTCTGAAACGGCAAGCTAAAAC-3′ and SynX6(r) 5′-CCGGTCGACGACCGTCTGAAACGGTGCGAGTATCTC-3′. PCR was carried out in 50 µl reaction volumes (45 µl Platinum Blue PCR Supermix (Invitrogen), 2 µl genomic DNA, 1.5 µl each primer). The PCR conditions were as follows: an initial step of 94°C for 5 min, followed by 30 cycles of 94°C for 45 sec, 55°C for 45 sec, 72°C for 2 min, and a final step of 72°C for 10 min. The PCR products were analyzed on a 1% agarose gel.

### Antigens

LOS used in the bactericidal depletion assay was purified according to the method of Westphal and Jann [22]. Lipopolysaccharide (LPS) from Salmonella enteric serotype typhimurium was purchased from Sigma Aldrich (St. Louis, MO). The PorA and fHbp genes were cloned in pT7-MAT-Tag-Flag-1 vector (Sigma) which were then used to transform E. coli cells BL21(DE3). The transformed cells were grown in LB broth, induced with 1mM IPTG, the cells were lysed and the His-tagged proteins purified on Ni columns. Serogroup A meningococcal polysaccharide (MAPS) was produced by SynCo BioPartners, Amsterdam, Netherlands. Serogroup C meningococcal polysaccharide (MCPS) was from FioCruz, BioMaguinhos, Brazil. Serogroup W-135 and Y meningococcal polysaccharides (MWPS and MYPS) were from Chiron. Tetanus toxoid (TT) was obtained from Wyeth Vaccines and Serum Institute of India. 4-cyno-dimethylamino pyridium tetrafluoroborate (CDAP) was purchased from Sigma Chemical Company (St. Louis, MO).

### Spot Blotting/Colony Blotting

The phenotype of the vaccine strains and the bactericidal target strains for serogroup A, C, Y, W and X *N. meningitidis* were determined by colony blotting as previously described [17]. Spot blotting, a variation of colony blotting where a suspension of the culture is spotted onto nitrocellulose rather than lifting colonies directly from an agar plate, was used to determine the LOS immunotype of target strains from the other groups. Colony blotting was performed as previously described [23]. The following monoclonal antibodies were used in the blotting procedures to determine the capsular polysaccharide (serogroup), PorA (serosubtype), and LOS immunotype of the target strains: serogroup B capsular polysaccharide (mAb2-1-B); PorA P1.16 (mAb 3-1-P1.16), PorA P1.7 and P1.7-2 (mAb 3-1-P1.7), PorA P1.2 (mAb 3-1-P1.2), PorA P1.5 (mAb was obtained from NIBSC) and P1.4 (mAb MN20B9.34); L8 LOS (mAb 2-1-L8), L3,7 LOS (mAb 9-2-L37), L1 LOS (mAb 17-1-L1), L2 LOS (mAb 27-1-L2). The monoclonal antibodies Jar5 (fHbp1) and Jar11 (fHbp2) were obtained from Dr. Dan Granoff.

### NOMV Preparation

The serogroup B *N. meningitidis* vaccine strains (H44/76, NZ9547 and B16B6) were grown in liquid culture using modified Catlin’s Medium in which the individual amino acids were replaced by 1% casamino acids (Becton Dickenson, Franklin Lakes, NJ, USA ) and iron (ferric sulfate) was reduced to 10% of the normal level (0.5 mg/L) to induce expression of iron uptake proteins. No antibiotics were added to the growth medium. Bacterial cultures (1L), were grown in 2800 ml baffled Fernbach flasks at 37^o^C with rotary shaking at 160–180 RPM. NOMV were extracted from packed cells as previously described [24].

### Preparation of fHbp1, fHbp2 and Fusion Protein of Variant 1 and Variant 2 fHbp(1+2)

The gene of factor H-binding protein variant 1 (fHbp1.1) was amplified by PCR using strain H44/76 genomic DNA as a template and the oligos 5′-AAGCTTCCTCGAGTGAGCAGTGGAGGGGGTGGTGTCGCC-3′ (forward) and 5′-GGCGGGGAATTCACTTATTGCTTGGCGGCAAGGCCGAT-3′ (reverse). The gene of fHbp2.16 was amplified by PCR using strain 7608 genomic DNA as a template and the oligos 5′-AAGCTTCCTCGAGTGAGCAGTGGAGGCGGCGGTGTCGCC-3′ (forward) and 5′-GGCGGGGAATTCACTTACTACTGTTTGCCGGCGATGCC-3′ (reverse). The PCR products were cloned in the Xho1 - Ecor1 site of pT7-MAT-Tag-Flag-1 vector (Sigma) which were then used to transform E. coli cells BL21(DE3). The transformed cells were grown in LB broth, induced with 1mM IPTG, lysed and the His-tagged proteins purified on Ni columns.

For fHbp(1+2) fusion protein, first the gene of fHbp1.1 was amplified by PCR using strain H44/76 genomic DNA as a template and the oligos 5′-AAGCTTCCTCGAGTGAGCAGTGGAGGGGGTGGTGTCGCC-3′ (forward) and 5′-CGATCCGCCACCGCCAGAGCCACCTCCGCCTGAACCGCCTCCACCTTGCTTGGCAAGGCCGAT-3′ (reverse). The PCR product was reamplified using the oligos 5′-AAGCTTCCTCGAGTGAGCAGTGGAGGGGGTGGTGTCGCC-3′ (forward) and 5′-GCCGCCTCCTCTAGAACCGCCACCGCCAGAGCCACC-3′ (reverse). The gene of fHbp2.16 was amplified by PCR using strain 7608 genomic DNA as a template and the oligos 5′-GGTGGAGGCGGTTCAGGCGGAGGTGGCTCTGGCGGTGGCGGATCGGGAGGCGGCGGTGTCGCC-3′(forward) and 5′-GGCGGGGAATTCACTTACTACTGTTTGCCGGCGATGCC-3′ (reverse). The PCR product was reamplified using the oligos 5′-GGTGGCGGTTCTAGAGGAGGCGGCGGTGTCGCC-3′ (forward) and 5′-GGCGGGGAATTCACTTACTACTGTTTGCCGGCGATGCC-3′ (reverse). The fHbp1 PCR product was digested with Xho1 and Xba 1 while the fHbp2 PCR product was digested with Xba1 and EcoR1. The digested products were cloned into the Xho1 EcoR1 site of pT7-MAT-Tag-Flag-1 vector (Sigma) which were subsequently used to transform E. coli cells BL21 (DE3). The transformed cells were grown in LB broth, induced with 1mM IPTG, the cells were lysed and the His-tagged proteins purified on Ni columns.

### Preparation of PS-fHbp(1+2) and PS-TT Conjugates

Conjugation of polysaccharide to fHbp(1+2) fusion protein was carried out using CDAP conjugation method of Lee et al. [25] with modification. On ice, 10 µL PS (10 mg/mL; Mn A, C, W or Y) is mixed with 1 µL CDAP (100 mg/mL in acetonitrile). In a cold room, the PS solution was mixed with 1 µL triethylamine (TEA, 0.2 M). After 1.5–2 hours incubation at 4^o^C, 4 µL of 0.1 M phosphate buffer, pH 5.5 was added to the reaction mixture followed by addition of 0.1 mg fHbp or hydrazide-modified TT prepared according to reference [26]. The conjugation reaction proceeded overnight in the cold room with agitation. After dialysis against 50 mM NaCl, 10 mM HEPES, buffer, pH 7.2 at 4^o^C, the conjugation product was adjusted to (PS) = 0.2 mg/mL with dialysis buffer and stored at 4^o^C.

### High Performance Size-exclusion Liquid Chromatography (HPSEC)

Samples of proteins, polysaccharides and conjugate products (25 µL, 0.1 mg/mL) were applied to a Waters Ultrahydrogel 2000 column with 0.9% NaCl, 10 mM Tris (pH 7.2), 1 mM EDTA, at 0.5 mL/minute in a Dionex HPLC system using Chromelean software with a UV detector at 280 nm for monitoring protein signal, and a refractive index (RI) detector for detecting protein, polysaccharide and conjugate.

### Immunization of Animals

Vaccines were thawed and formulated at the desired concentration prior to vaccination. Groups of 10 CD-1 mice were vaccinated intraperitoneally with various combinations of antigens. In all experiments three doses of the antigens (0.1 mL per mouse) were administered at 0, 4 and 8 weeks and blood was taken at 0, 7, and 10 weeks following the initial vaccination. The concentration of NOMV used for immunization was 9 µgs of protein 0.1 mL, and the concentration of both the fusion protein and the capsular conjugates used for immunization were 1 µg in 0.1 ml each. Data reported were obtained using the final (10 week) sera.

### Serological Assays

The serum bactericidal assay was performed as previously described [27] using normal human serum prescreened for lack of intrinsic bactericidal activity against the test strain as a source of complement. The pooled mouse sera were heat inactivated and tested using a starting dilution of 1∶2. The reciprocal of the highest dilution of serum that killed ≥50% of the bacteria was taken as the end point titer of the serum. If there was less than 50% killing at a dilution of 1∶2, the serum was assigned a titer of 1∶1. The highest dilution tested was 1∶512. All the pre-vaccination sera from the mice lacked bactericidal antibodies against any of the test strains and were assigned a titer of 1∶1. The bactericidal depletion assay was performed as described [27] by coating wells of a 96-well microplate with 100 µgs of antigens to be tested, then blocking, and washing the wells. The sera to be tested were diluted to the 50% kill endpoint and incubated in the coated wells for 4 hours, after which the sera were transferred to a fresh plate and tested for residual bactericidal activity. The inhibition was calculated by comparing the bactericidal activity of the unabsorbed sera (100% killing, 0% inhibition) to that of sera absorbed with antigens before the bactericidal assay. Whole cells (wild-type vaccine strains) served as a positive control since they absorbed all the bactericidal antibodies whereas BSA and Salmonella LPS served as negative controls absorbing very little (<5%) of the bactericidal antibodies from the test sera.

### Measurement of Endotoxicity by Whole Blood Cytokine Release Assay

The endotoxic activity of the NOMV was measured using the whole blood cytokine release assay as previously described [28]. The two cytokines measured in the assay were IL-6 and TNF- α.

## Results

### Characterization of the Vaccine Strains and the NOMV Vaccine Derived from the Three Strains

**Figure 1:**
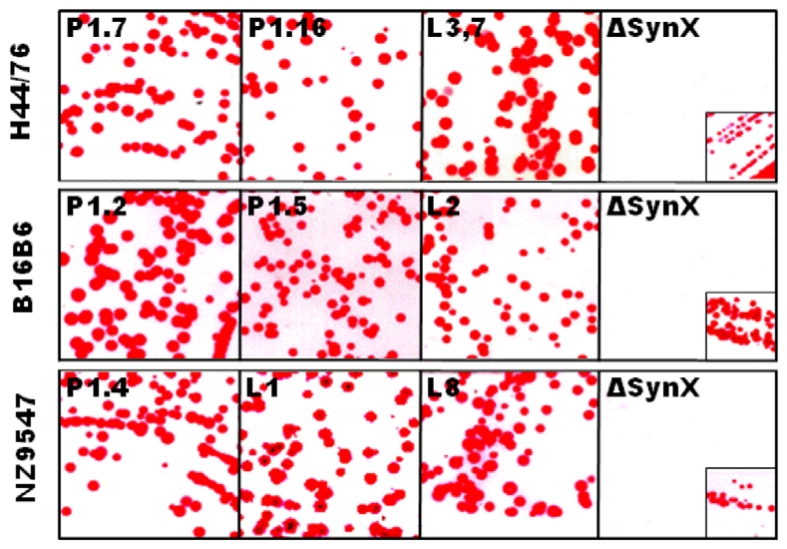
Capsule, LOS and PorA expression by the Vaccine Strains used to make the NOMV. Colony blots of the vaccine strains with monoclonal antibodies specific for the LOS and PorA antigenic epitopes. The monoclonal antibodies used to detect the capsule, PorA and LOS are described in Materials and Methods. The insert in each of the capsular frame shows the result with a positive control.

**Figure 2:**
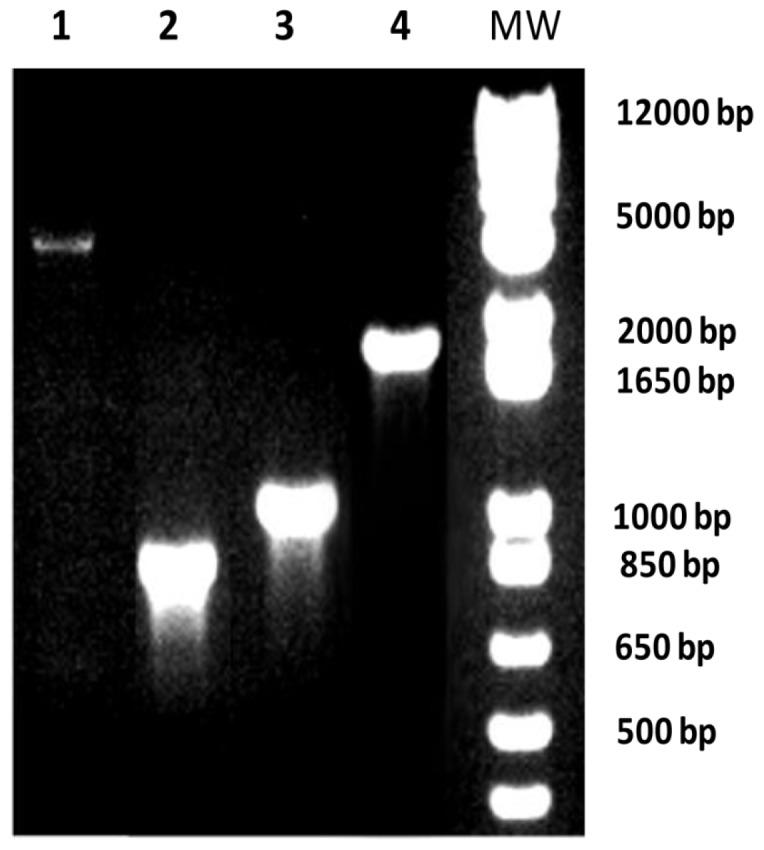
Analysis of the *lpxL1 and synX* gene PCR products from wild type and vaccine strains. *lpxL1 and synX* gene PCR products obtained from the amplification of wild type and mutant (*ΔlpxL1ΔsynX*) NZ9547 *N. meningitidis* strain genomic DNA. The *lpxL1* gene PCR products obtained from the amplification of mutant and wild type genomic DNAs are shown in lanes 1 and 2 respectively. The *synX* gene PCR products obtained from the amplification of wild type and mutant genomic DNA are shown in lanes 3 and 4 respectively.

**Figure 3:**
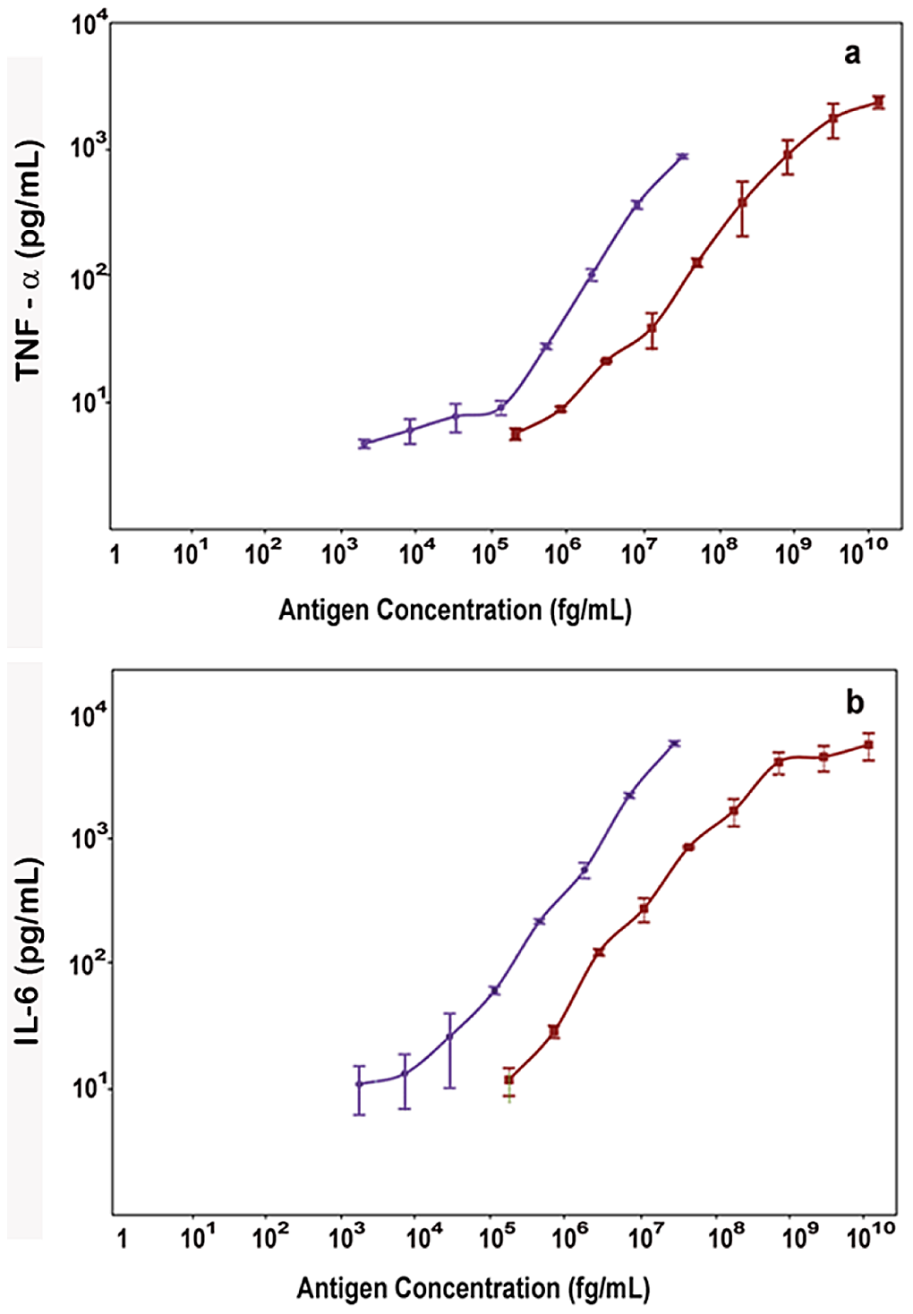
Measurement of the endotoxin activity of the NOMV vaccine. Measurement of endotoxin activity of the NOMV vaccine by the whole blood cytokine release assay using TNF- α and IL-6 as indicators. In Fig. 3a, the induction of TNF-α by the wild-type H44/76 NOMV (*circles*) and (*ΔlpxL1ΔsynX*) experimental trivalent NOMV vaccine (*squares*) is shown. In Fig. 3b, the induction of IL-6 by the wild-type H44/76 NOMV (*circles*) and (*ΔlpxL1ΔsynX*) experimental trivalent NOMV vaccine (*squares*) is shown.

**Figure 4:**
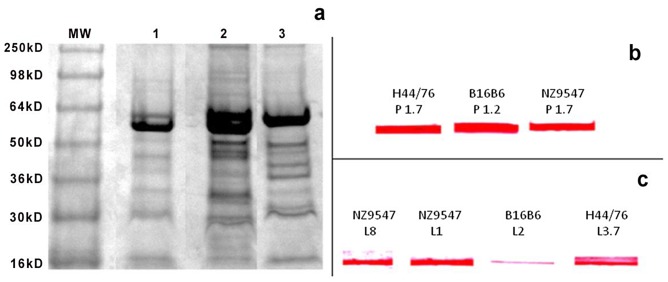
Analysis of the major surface antigens in the vaccine NOMV. SDS-PAGE gel (10%) of NOMV obtained from each of the three strains are shown in Fig. 4a. Comassie Blue stained gel showing NOMV from H44/76 in lane 1, NOMV from B16B6in lane 2 and NOMV from NZ9547 in lane 3. In Fig. 4b is shown the results of the western blot indicating the presence of PorAs in the NOMV from the three strains, and in Fig. 4c is shown the results of the Western blots indicating the presence of LOS in the NOMV from the three strains.

The phenotypic characterization of the three serogroup B *N. meningitidis* strains H44/76, B16B6 and NZ9547 by colony blotting for the serosubtype (PorA) and immunotype (LOS) is shown in Figure 1. The data in Figure 1 show that the strain H44/76 expresses the P1.7,16 PorA and the L3,7 LOS. The vaccine strain B16B6 expresses the P1.2,5 PorA and the L2 LOS. The vaccine strain NZ9547 expresses P1.7–2,4 PorA and the L1 and L8 LOS. The serosubtypes (PorA) for the three vaccine strains were confirmed by sequencing [18]. Figure 1 also shows the absence of the capsule in all three *N. meningitidis* strains used to make the experimental vaccine. This result was confirmed by PCR shown in Figure 2, where the *synX* gene was knocked out by the insertion of the Kan^r^ gene resulting in the generation of a larger (2 Kb) PCR fragment from the capsule negative vaccine strain when compared to the smaller (1 kb) PCR fragment obtained from the capsulated wild-type strain. Also shown in Figure 2, is the *lpxL1* knockout by the insertion of the tet^r^ gene which resulted in the generation of a 3.5 kb fragment from the vaccine strains by PCR, as compared to the smaller (0.95 kb) fragment obtained from the wild-type strains. The *lpxL1* knockout results in the conversion of the toxic hexa-acylated LOS to the relatively less toxic penta-acylated LOS variant without affecting the immunogenicity of the LOS. Figure 3 shows the results of the whole blood cytokine release assays for the NOMV vaccine and the NOMV obtained from H44/76 wild-type parental strain. The results show that the NOMV from the vaccine (which contains the *lpxL1* deletion) is between 10 and 100 fold less toxic than the parental H44/76 NOMV based on the induction of TNF- α and IL-6. This is consistent with our earlier observations that NOMVs containing penta-acylated LOS are less endotoxic than the wild type hexa-acylated LOS variant [28]. Furthermore, NOMV containing the penta-acylated variant were not found to be toxic when administered intramuscularly in humans [29]. Figure 4a shows the results of the SDS-PAGE gel of the NOMV from the three strains used for the immunogenicity studies. The presence of the two major outer membrane antigens, LOS and PorA from each of the three strains in the NOMV vaccine was confirmed by Western blot (Fig. 4b and Fig. 4c).

### Bactericidal Activity against Serogroup B N. meningitidis Strains

Since the NOMVs were obtained from serogroup B *N. meningitidis* strains, the first set of target strains analyzed for killing by the bactericidal activity of the immunized mouse sera were the wild type parents of the vaccine strains and a diverse panel of serogroup B strains taken from the repository of case strains at the Walter Reed Army Institute of Research. The strains in the panel share either LOS and or PorA with the vaccine strains. An immunizing dose of 9 µg per mouse was used since earlier studies showed this to be optimal in inducing a good bactericidal response against a diverse panel of serogroup B *N. meningitidis* strains [17]. The results in Table 1 show that the bactericidal titer for all the serogroup B *N. meningitidis* strains was greater than 1∶4 with the exception of *N. meningitidis* serogroup B strain 6557. The reason for the resistance of *N. meninigitidis* serogroup B strain 6557 is unclear since it does share LOS with the vaccine strains. The results obtained in this study are similar to results obtained using a completely different NOMV vaccine that was extensively genetically modified for both safety and increased immunogenicity purposes [17].

**Table 1:** Bactericidal activity against serogroup B *N. meningitidis* strains.

Strain	MLST/Clonal complex	PorA	LOS	Antigens shared withthe vaccine strains	Titer
H44/76	ST-32 complex/ET-5 complex	P1.7,16	L3,7	vaccine strains	16
8570	ST-32 complex/ET-5 complex	P1.19,15	L3,7	LOS	256
7608	ST-8 complex/cluster A4	P1.5-1,2-2	L4		64
8047	ST-8 complex/cluster A4	P1.5-1,2-2	L3,4,7	LOS	64
NZ 9547	ST-41/44 complex/lineage 3	P1.7-2,4	L1,8	vaccine strains	128
B16B6	ST-11 complex/ET-37 complex	P1.5,2	L2	vaccine strains	512
6557	ST-41/44 complex/lineage 3	P1.22-1,14	L1,3,7	LOS	2
190I	ST-41/44 complex/lineage 3	P1.18,25	L1,3,7	LOS	128
6275	ST-41/44 complex/lineage 3	P1.5,2	L3,7	LOS and PorA	512
M1080	ST-41/44 complex/lineage 3	P1.7-1,1	L1,3,7	LOS	8
9162	ST-32 complex/ET-5 complex	P1.7-2,3	L3,7	LOS	128
99M	ST-11 complex/ET-37 complex	P1.5,2	L3,7	LOS and PorA	512

Pooled sera from mice immunized with the NOMV vaccine were tested for bactericidal activity with human complement against the serogroup B *N. meningitidis* strains listed. The data are reciprocal titers. The MLST, PorA, and LOS (immunotype) of the target strains and antigens shared with the vaccine strains are also shown in the Table.

### Bactericidal Activity Induced by PorA and LOS in the Experimental Vaccine

**Figure 5:**
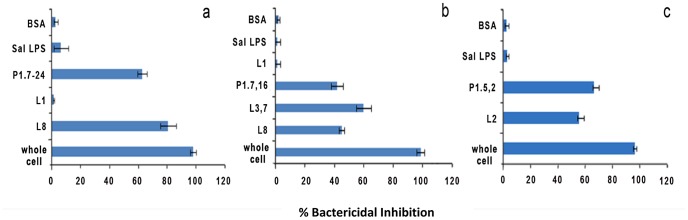
Characterization antibody specificity by the bactericidal Inhibition assay. Pooled sera from mice immunized with the NOMV vaccine was analyzed for bactericidal antibody specificity using *N. meningitidis* wild type vaccine strains as the target strain in a bactericidal depletion assay. In Fig. 5a the bactericidal target strain used was wild type NZ9547, in Fig. 5b the bactericidal target strain used was wild type H44/76, and in Fig. 5c the bactericidal target strain used was wild type B16B6. The antigens used to for depletion are shown in the figure.

To study the role of PorA and LOS in the vaccine preparation, the three wild type parental strains were used as targets in the bactericidal inhibition assay as described in Materials and Methods. As expected, bactericidal antibodies present in the sera of mice immunized with the experimental trivalent vaccine were completely depleted when pre-adsorbed by whole cells (H44/76, NZ9547 and B16B6) before being used in the bactericidal assay (Fig. 5). The depletion of bactericidal antibodies in the sera of mice immunized with the experimental trivalent vaccine by the controls (BSA and LPS from *Salmonella enterica*) was minimal. Interestingly, the bactericidal inhibition assay showed that most of the antibody directed against the target strain NZ9547 was to the surface antigens PorA (P1.7–2,4) and L8 LOS (Fig. 5a), but not the L1 LOS that is also present on the surface of the target strain. The reason why L1 LOS did not induce a bactericidal response is unclear. The bactericidal inhibition assay also showed that most of the antibody directed against the target strain H44/76 was to the surface antigens PorA (P1.7,16) and L3,7 LOS (Fig. 5b). Since L3,7 LOS has an L8 core, the depletion seen with the L8 LOS can be attributed to the presence of cross reacting antibodies induced by L3,7 present in the vaccine. The bactericidal inhibition assay showed that most of the antibody directed against the target strain B16B6 was to the surface antigens PorA (P1.5,2) and the L2 LOS (Fig. 5c). The data obtained from the bactericidal inhibition assay shows that most of the bactericidal antibody is directed to the two dominant surface antigens LOS and PorA.

### Bactericidal Activity against non serogroup B N. meningitidis Strains

The bactericidal activity of the pooled mouse sera was tested on a limited number of strains from serogroups A, C, Y, W and X in the WRAIR collection for which human complement was available. The phenotype (PorA and LOS) and genotype (MLST) of the strains in this panel are shown in Table 2. As seen in Table 2, the strains in serogroup A were highly resistant to killing in the bactericidal assay. Since the serogroup A strains do not share the major outer membrane antigens such as PorA and LOS with the vaccine strains, it is not surprising that the experimental trivalent vesicle vaccine was unable to induce a protective response to these strains.

**Table 2:** Bactericidal activity against non serogroup B *N. meningitidis* strains.

Strain	Group	MLST/Clonal complex	PorA	LOS	Antigens shared withthe vaccine strains	Titer
5878	A	ST-4 complex/subgroup IV	P1.7,13-1	L9	none	2
8991	A	ST-5 complex/subgroup III	P1.20,9	L8,9	none	2
7891	A	ST-5 complex/subgroup III	P1.20,9	L11	none	1
8822	A	ST-1 complex/subgroup I/II	P1.5-1,2-2	L10	none	1
8837	C	ST- 41/44 complex/lineage 3	P1.19,15	L3	LOS	2
5416	C	ST-11 complex/ET-37 complex	P1.5,2-1	L3	LOS	512
5660	C	ST-11 complex/ET-37 complex	P1.5,2-1	L3	LOS	128
7510	W	ST-178 complex	P1.19,15	L3,7	LOS	>512
8020	Y	ST-23 complex/Cluster A3	P1.5-1,2-2	L3	LOS	>512
9463	Y	ST-167 complex	P1.5-1,10-4	L3-5,7-5	LOS	>512
9557	X	ST-5403	P1.19,26	L8	LOS	32
9558	X	ST-5403	P1.19,26	L8	LOS	32
9559	X	ST-5403	P1.19,26	L8	LOS	32

Pooled sera from mice immunized with the NOMV vaccine were tested for bactericidal activity with human complement against the non-serogroup B *N. meningitidis* strains listed. The data are reciprocal titers. The MLST, PorA, and LOS (immunotype) of the target strains and antigens shared with the vaccine strains are also shown in the Table.

All three serogroup C strains in the panel share the major outer membrane antigen, L3 LOS, with the vaccine strains. Furthermore, two of the three strains also express the P1.5 PorA epitope that is also expressed by one of the vaccine strain (B16B6). As seen in Table 2, the two serogroup C strains (5416 and 5660) that share both the LOS and PorA were killed in the bactericidal assay with titers of 512 and 128 respectively. Interestingly, the two serogroup C strains (5416 and 5660) and the vaccine strain B16B6 belong to the ST11 complex. Thus it is quite possible that vaccine strain B16B6 and the two serogroup C strains 5416 and 5660 share other surface antigens besides LOS and PorA. The serogroup C strain 8837 appear to be resistant despite sharing the LOS with the vaccine strains and being part of the ST41/44 complex like the vaccine strain NZ9547. The reason for the resistance to killing in the bactericidal assay are not clear since anti-L3,7 antibodies are induced by the experimental trivalent vesicle vaccine.

The three strains in serogroups W and Y share the major outer membrane antigen LOS with the vaccine strain, and, as seen in Table 2, all three strains are effectively killed in the bactericidal assay at titers greater than 512. The three serogroup X strains were independent isolates from an outbreak in Western Kenya. Since all three strains were part of a single outbreak and belong to ST 5403, they are considered the same clone, and any observable differences between the independent isolates could be attributed to clonal variation. They share the major outer membrane antigen, L8 LOS with the vaccine strain, and as seen in Table 2, are killed in the bactericidal assay at titers of 32. Taken together, these results suggest that a combination of NOMV and serogroup A polysaccharide (MAPS) would result in a vaccine that protects against serogroups A, B, C, W, X and Y *N. meningitidis* infections.

### Preparation and Characterization of PS-fHbp(1+2) Conjugate

**Figure 6:**
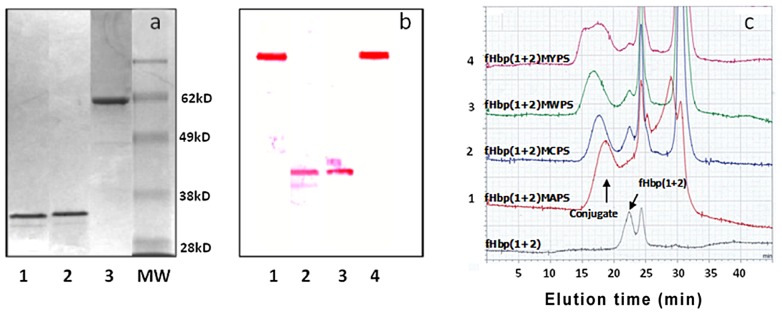
Analysis of purified fHbp variants and fHbp capsular conjugates. In Fig. 6a, is the Commassie Blue stained gel (10%) showing purified fHbp1 (Lane 1), fHbp2 (Lane 2) and fHbp(1+2) fusion product (Lane 3). In Fig. 6b is the Western blot analysis using mAbs Jar 5 (specific for fHbp1) and Jar 11 (specific for fHbp2); fHbp(1+2) in lanes 1 and 4 developed with mAbs Jar 5 and Jar 11 respectively; fHbp1 in lane 2 developed with mAb Jar 5, and fHbp2 in lane 3 developed with mAb Jar 11. In Fig. 6c are shown the HPLC profiles of fHbp(1+2) and the conjugate products fHbp(1+2)MAPS, fHbp(1+2)MCPS, fHbp(1+2)MWPS, fHbp(1+2)MYPS monitored at 280 nm.

Since vaccines based on fHbp1 and fHbp2 have also been shown to induce broad protection, the utility of using a fusion protein made of fHbp1 and fHbp2 to act as a protein carrier for *N. meningitidis* capsular polysaccharide was investigated. The fusion of fHbp1and fHbp2 DNA, the expression of the fusion protein and its purification is described in Materials and Methods. An SDS-PAGE gel of the purified fHbp1, fHbp2 and fHbp(1+2) fusion proteins is shown in Fig. 6a. As expected, the fusion protein fHbp(1+2) is much larger than either of the two purified protein. As shown in Fig. 6b, the fusion protein also reacts to the two antibodies Jar 5 (specific for fHbp1) and Jar 11(specific for fHbp2). The purified fHbp(1+2) fusion protein was conjugated to *N. meningitidis* PS as described in Materials and Methods. The size-exclusion HPLC profiles of fHbp(1+2) conjugated to MAPS, MCPS, MWPS and MYPS are shown in Fig. 6c. The peak of fHbp(1+2) is at minute 23 as seen in Fig. 6c. Conjugate formation between PS and the protein is indicated by the shift of protein signal from minute 23 to the higher molecular weight position at minutes 15–19 upon conjugation with, the residual unconjugated protein unmoved.

### Bactericidal Activity Induced by PS-fHbp(1+2) Conjugates

As shown in Table 3, immunization with fHbp(1+2) fusion protein, which is a combination of fHbp1.1 and fHbp2.16, did induce a four-fold or greater increase in bactericidal antibodies against strains 8570 (serogroup B), strain 7510 (serogroup W), strain 9557 (serogroup X) and strains 8020 and 9463 (serogroup Y). With the exception of 8570 (serogroup B) all of these other strains that were killed expressed alleles of fHbp that differed from the vaccine. The strains that were resistant to killing presumably did not express enough fHbp on the surface for the antibodies to be bactericidal. Immunization with The fHbp (1+2) all capsular conjugates did indeed induce specific bactericidal antibodies that killed serogroup A, C, W and Y strains (Table 3). Sera from mice immunized with fHbp(1+2) were not tested for bactericidal activity against serogroup B strains since they have a different capsule and LOS is better than fHbp (1+2) at inducing protective antibodies (Tables 2 and 3). The data shows that fHbp can serve as a carrier for the *N. meningitidis* capsular polysaccharides (Table 3). The data also shows that anti-fHbp bactericidal antibodies are induced upon immunization with fHbp(1+2) all capsular conjugates as evidenced by the killing of serogroup X strains at low titers. Immunization with serogroup A capsular conjugate, MAPS-fHbp(1+2) resulted in the killing of serogoup A strains at high titers because of the induction of anti-capsular bactericidal antibodies. Interestingly, the killing of 2 of 3 serogroup X strains at low titers in comparison to serogroup A strains when immunized with MAPS-fHbp(1+2) capsular conjugate, could be attributed to the induction of anti-fHbp antibodies. Thus the results suggest that the MAPS-fHbp(1+2) capsular conjugate may serve as a good vaccine candidate for the sub-Saharan meningitis belt where strains from these two serogroups are mainly responsible for the meningitis epidemics. It is also possible that the immunogenicity of these conjugates could be further enhanced with adjuvants.

**Table 3:** Bactericidal activity induced by PS-fHbp(1+2) conjugates.

				Immunogen		
				fHbp(1+2) fusion protein	all PS-fHbp(1+2) capsular conjugate*	MAPS-fHbp(1+2) capsular conjugate
Strain	MLST/Clonal Complex	Serogroup	fHbp	Titer	Titer	Titer
H44/76	ST-32 complex/ET-5 complex	B	1.1	1	ND	ND
8570	ST-32 complex/ET-5 complex	B	1.1	4	ND	ND
NZ 9547	ST-41/44 complex/lineage 3	B	2.64	1	ND	ND
B16B6	ST-11 complex/ET-37 complex	B	2.22	1	ND	ND
5878	ST-4 complex/subgroup IV	A	1.5	1	>512	>512
8991	ST-5 complex/subgroup IV	A	1.5	1	>512	>512
7891	ST-5 complex/subgroup III	A	1,5	1	256	>512
8822	ST-1 complex/subgroup I/II	A	1.4	1	>512	>512
8837	ST-41/44 complex/lineage 3	C	2.72	1	512	ND
5416	ST-11 complex/ET-37 complex	C	2.22	1	256	ND
5660	ST-11 complex/ET-37 complex	C	2.22	1	128	ND
7510	ST-178 complex	W	1.34	128	64	ND
8020	ST-23 complex/cluster A3	Y	2.25	>512	>512	ND
9463	ST-167 complex	Y	2.23	>512	>512	ND
9557	ST-5403	X	1.61	4	4	16
9558	ST-5403	X	1.61	2	4	8
9559	ST-5403	X	1.61	2	4	1

* The *N. menigitidis* capsular polysaccharides used were from serogroups A, C, W, and Y.

ND = not determined.

Pooled sera from mice immunized with fHbp(1+2) and fHbp(1+2) capsular conjugates were tested for bactericidal activity with human complement against the *N. meningitidis* strains from serogroups A, B, C, W, Y and X.

### Bactericidal Activity of a Mixture of NOMV and Either MAPS-fHbp(1+2) or MAPS-tetanus Toxoid Conjugate

Mice immunized with either NOMV or a mixture of NOMV and fHbp (1+2) fusion protein were able to protect against serogroup B *N. meningitidis* strains, but were not able to protect against serogroup A *N. meningitidis* strains (Table 4). Table 4 also shows that the fHbp(1+2) fusion protein alone did not induce much bactericidal antibody and the MAPS-fHbp(1+2) conjugate did not induce bactericidal titers superior to MAPS-tetanus toxoid conjugate. However, the fusion protein did provide protection against *N. meningitidis* strains from serogroups Y and W (Table 3). On the other hand, mice immunized with a mixture of NOMV and MAPS-fHbp(1+2) conjugate or MAPS-tetanus toxoid conjugate produced bactericidal antibodies that were protective against not only serogroup B but also serogroup A *N. meningitidis* strains. Since the NOMV have been shown to protect against serogroups B, C, W, Y and X, but not serogroup A, one can conclude from the data shown in Tables 2 and 4 that a mixture of NOMV and a capsular- protein conjugate could induce protection against all major *N. meningitidis* serogroups.

**Table 4:** Bactericidal activity of NOMV and either MAPS-fHbp(1+2) or MAPS-tetanus toxoid conjugate.

		Immunogen			
		fHbp(1+2) fusion protein	Trivalent NOMV Vaccine plus fHbp(1+2) fusion protein	Trivalent NOMV Vaccine plus MAPS-fHbp(1+2) capsular conjugate	Trivalent NOMV Vaccine plus MAPS-tetanus toxoid capsular conjugate
Strain	Group	Titer	Titer	Titer	Titer
H44/76	B	1	32	16	32
8570	B	4	256	256	512
7608	B	1	128	64	128
8047	B	1	64	64	256
NZ9547	B	1	256	256	512
B16B6	B	1	512	256	512
6557	B	1	16	2	2
190I	B	1	128	256	256
6275	B	1	>512	>512	>512
M1080	B	1	32	32	32
9162	B	2	128	128	128
99M	B	2	512	>512	512
5878	A	1	2	>512	>512
8991	A	1	2	>512	>512
7891	A	1	1	128	512
8822	A	1	1	>512	>512

Pooled sera from mice immunized with immunogens shown in the table and tested for bactericidal activity with human complement against the *N. meningitidis* strains from serogroups A and B.

## Discussion

To date we do not have a single licensed vaccine to prevent disease against all *N. meningitidis* serogroups. Licensed capsular conjugate vaccines are available for individual *N. meningitidis* serogroups A, C, W and Y. In the case of *N. meningitidis* serogroup B, while several subcapsular vaccine preparations are currently being studied for their protective efficacy, only the 4CMenB vaccine has progressed to licensing in Europe. Cost-effectiveness modeling of new meningococcal vaccines in England suggests that, given current disease levels, it is unlikely that these vaccines would be cost-effective [30]. Thus there is a need for a vaccine against all the disease serogroups of *N. meningitidis*, that is also cost-effective.

fHbp which is present in both 4CMenB and the bivalent fHbp vaccine has been shown to be protective against other *N. meningitidis* serogroups [19]. The recently described NOMV vaccine has also been shown to be effective against other *N meningitidis* serogroups [18]. Further investigation on the vaccine revealed that LOS and PorA appear to be the major inducers of the bactericidal response [17], [31], of which the LOS appears to be broadly cross-protective. Based on those observations coupled with the observation that vaccines containing L2 and L3,7 LOS would possibly cover 80% of the pathogenic serogroup B *N. meningitidis* strains [20], and that approximately 60% of the disease causing serogroup B *N. meningitidis* strains in the MLST database belong to either ST 41/44, ST-11 or ST32 MLST complex [21], we developed a simple NOMV vaccine from strains H44/76, NZ9547 and B16B6 that contains the endogenous PorAs and LOS. The only modification to the strains were the knocking out of the capsule (*ΔsynX*) and detoxifying the LOS (*ΔlpxL1*). This vaccine differs significantly from the previously described NOMV vaccine [17] since it does not express additional PorAs other than the endogenous PorAs and does not over express minor surface antigens such as fHbp and the phase variant NadA. Instead, in this vaccine the capsular polysaccharides are conjugated to fHbp which is then added to the NOMV to make it a multiple serogroups *N. meningitidis* vaccine. NOMV vaccines that are *ΔlpxL1ΔsynX* have been shown to be safe and induce a good antibody response to both PorA and LOS in humans. This newly developed vaccine induced bactericidal antibodies that were specific to both PorA and LOS. This NOMV vaccine as expected induced broad cross protection against *N. meningitidis* strains not only of serogroup B, but also serogroups C, W, Y and X. Since fHbp was shown to be protective against multiple serogroups [19], it was thought that conjugating fHbp to capsular polysaccharide and combining it with NOMV would result in a vaccine formulation that would be protective against most disease causing strains of *N. meningitidis*. The fHbp conjugates by themselves were capable of inducing a protective response against serogroups A, C, W, Y and X. Interestingly, the serogroup A polysaccharide fHbp conjugate induced a protective response against both serogroup A and also serogroup X *N. meningitidis* strains, which make it a good vaccine candidate for the sub-Saharan meningitis belt. The protection induced by fHbp or the fHbp capsular conjugates against serogroup B was not impressive despite the presence of bactericidal antibodies to both fHbp1 and fHbp2. This suggests that a critical threshold of fHbp expression is needed on the surface for anti-fHbp antibodies to be effective. The fact that fHbp was a good carrier for the capsular polysaccharide suggested that a combination of the NOMV vaccine along with the fHbp capsular conjugates would result in a vaccine that would be protective against the major disease causing serogroups of *N. meningitidis*. The data in this study shows that a combination of the NOMV along with serogroup A capsular polysaccharide conjugated to fHbp or tetanus toxoid was sufficient to induce a protective response against most *N. meningitidis* strains. Recently, other investigators have shown that immunization with a combination of NOMV-fHbp and MenA polysaccharide would be sufficient for the prevention of meningococcal infections [32], where fHbp is mainly responsible for the protection against the various serogroups tested and the resistant strains from serogroup A being protected by MenA polysaccharide conjugate in the vaccine preparation.

While it may be possible to protect against most N. meningitidis infections by using a combination of NOMV and MenA polysaccharide as an immunogen, given the safety and efficacy of the quadrivalent polysaccharide a combination of NOMV derived from 2 strains expressing L2 and either L3,7 or L8 along with capsules from serogroups A, C, W-135 and Y conjugated to either fHbp(1+2) or tetanus toxoid may be both protective against most *N. meningitidis* infections, and also a safer option. The use of NOMV is crucial for the induction of bactericidal antibodies to LOS since deoxycholate extracts of the outer membrane of *N. meningitidis* strains used as vaccines fail to induce bactericidal antibodies to LOS [33]. While the panel of strains used in this study is fairly limited, the data provide a reason to further investigate the potential of such combination vaccines. Vaccines combining capsular and sub-capsular antigens may also be effective against pneumococcal disease where capsular polysaccharide vaccine usage has resulted in increased incidence of disease due to capsule types not present in the vaccine [34]–[36].
